# Supplementary figures and images for: Identification of CDRH3 loops in the B cell receptor repertoire that can be engaged by candidate immunogens
Source: PLoS Pathog. 2023 May 17;19(5):e1011401. doi: 10.1371/journal.ppat.1011401 (PMC10228794; doi:10.1371/journal.ppat.1011401)

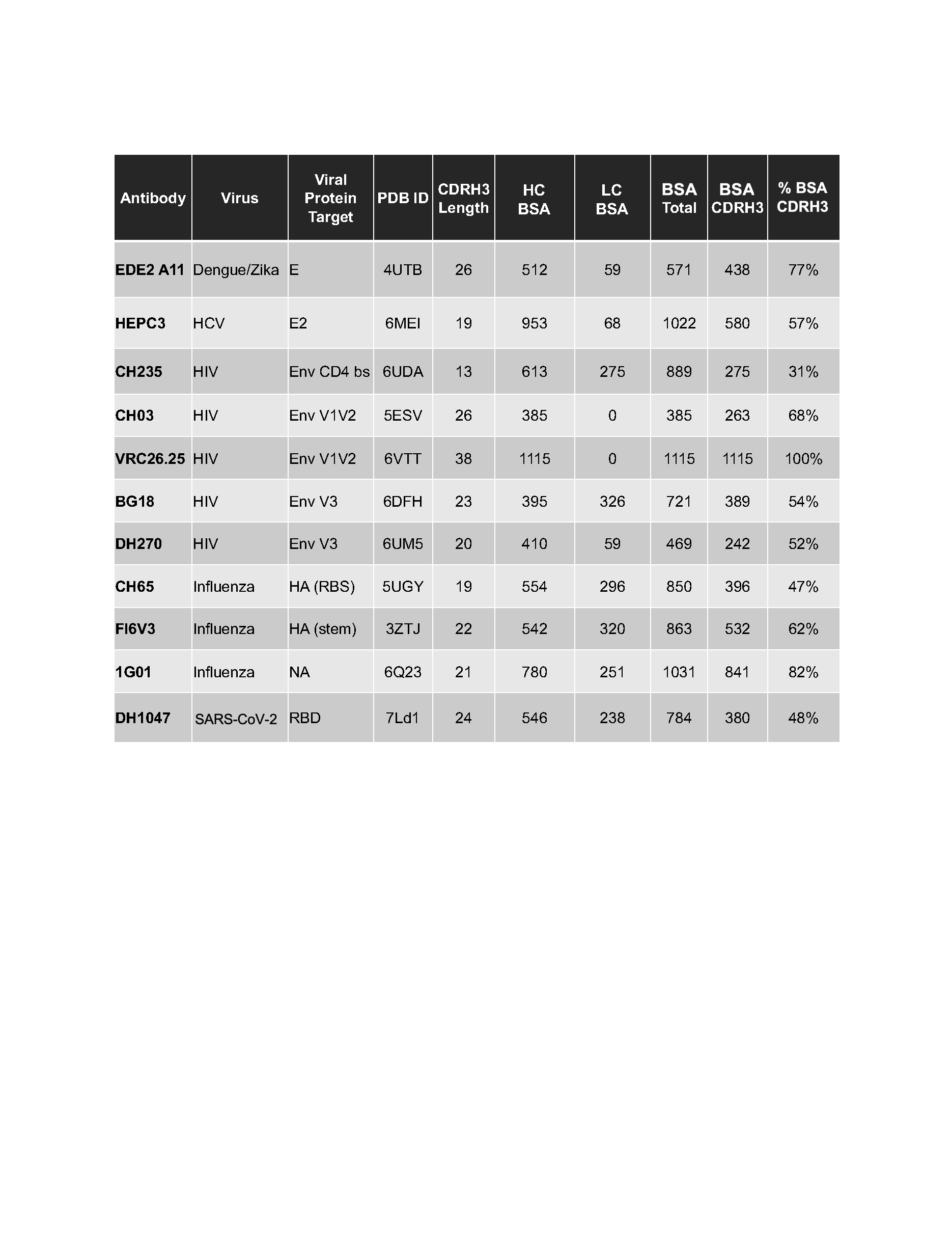

Supplement: S1 Table — Antibody/antigen structural complexes describing a wide variety of antibody bound to viral proteins were analyzed using PDBePISA to determine the buried surface area (BSA) at the binding interface in units of Å2. BSA binding contributions for residues in the light chain (LC) heavy chain (HC), and CDRH3 loop are reported for each antibody. (TIFF) [file ppat.1011401.s001.tiff]

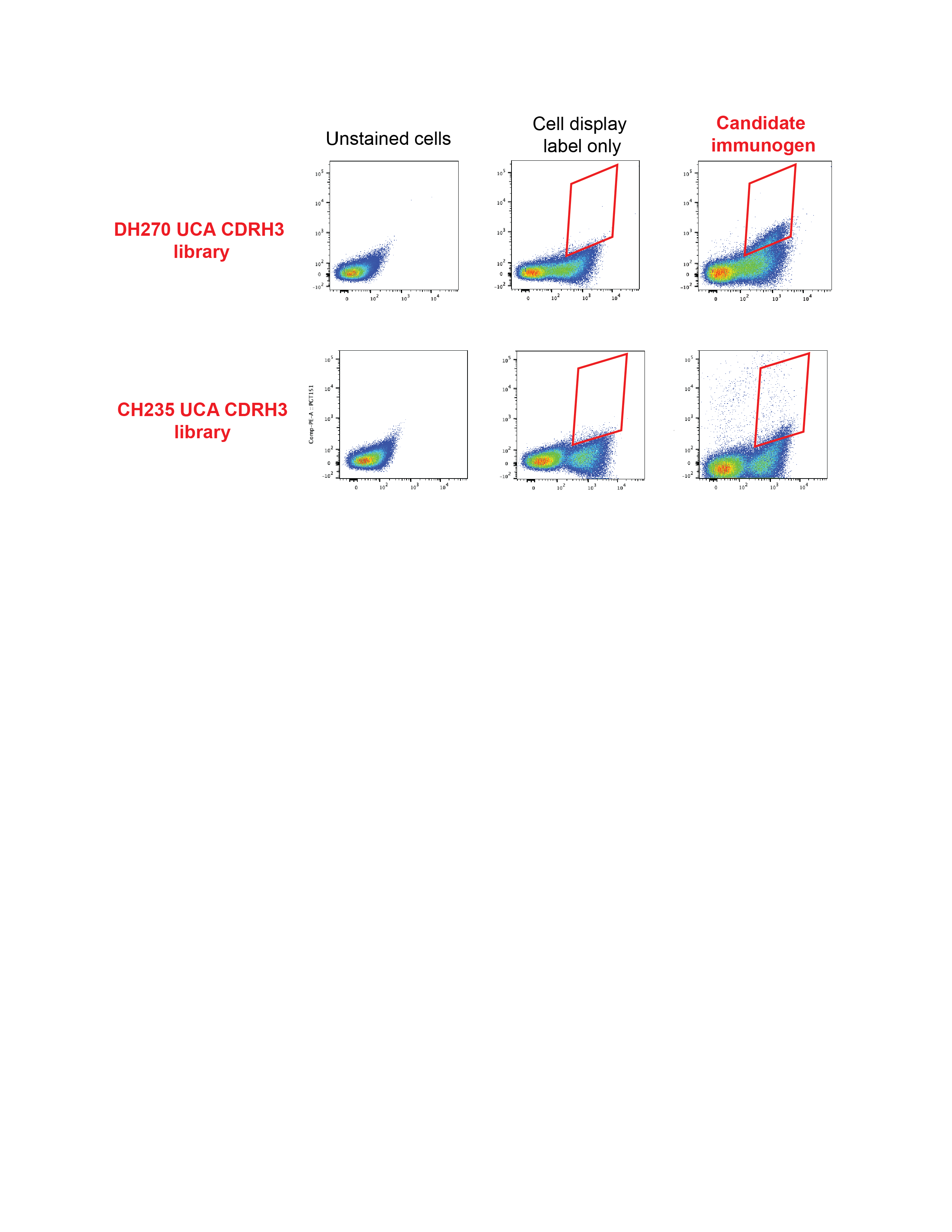

Supplement: S1 Fig — 10.17DT and CH505.M5.G458Y scFv libraries were displayed on the surface of yeast and labeled for cell surface display (x-axis) and binding to the candidate immunogen (y-axis). DH270 UCA and CH235 UCA libraries were sorted with 300nM concentration of biotinylated SOSIP target immunogens 10.17DT and CH505.M5.G458Y respectively, and all binding clones were recovered for downstream DNA isolation and NGS analysis. (TIFF) [file ppat.1011401.s003.tiff]

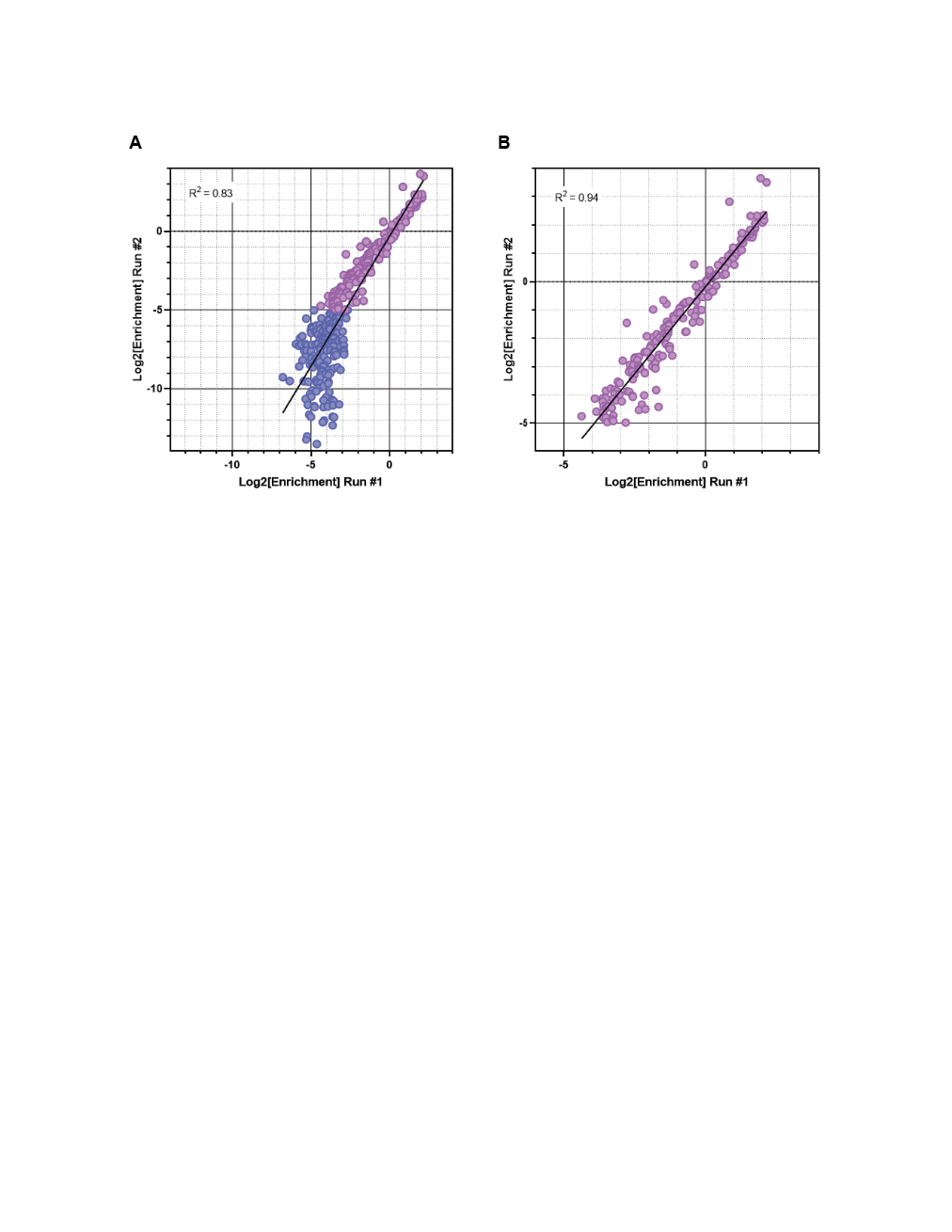

Supplement: S2 Fig — A. Enrichment scores of two sort replicates completed on separate day are shown. The correlation was strongest for enrichment scores higher than -5 (pink) and decreased somewhat for values below this threshold (purple). An enrichment score of 5 corresponds to a 32-fold depletion of a particular mutation from the initial library upon sorting with 10.17DT. (TIFF) [file ppat.1011401.s004.tiff]

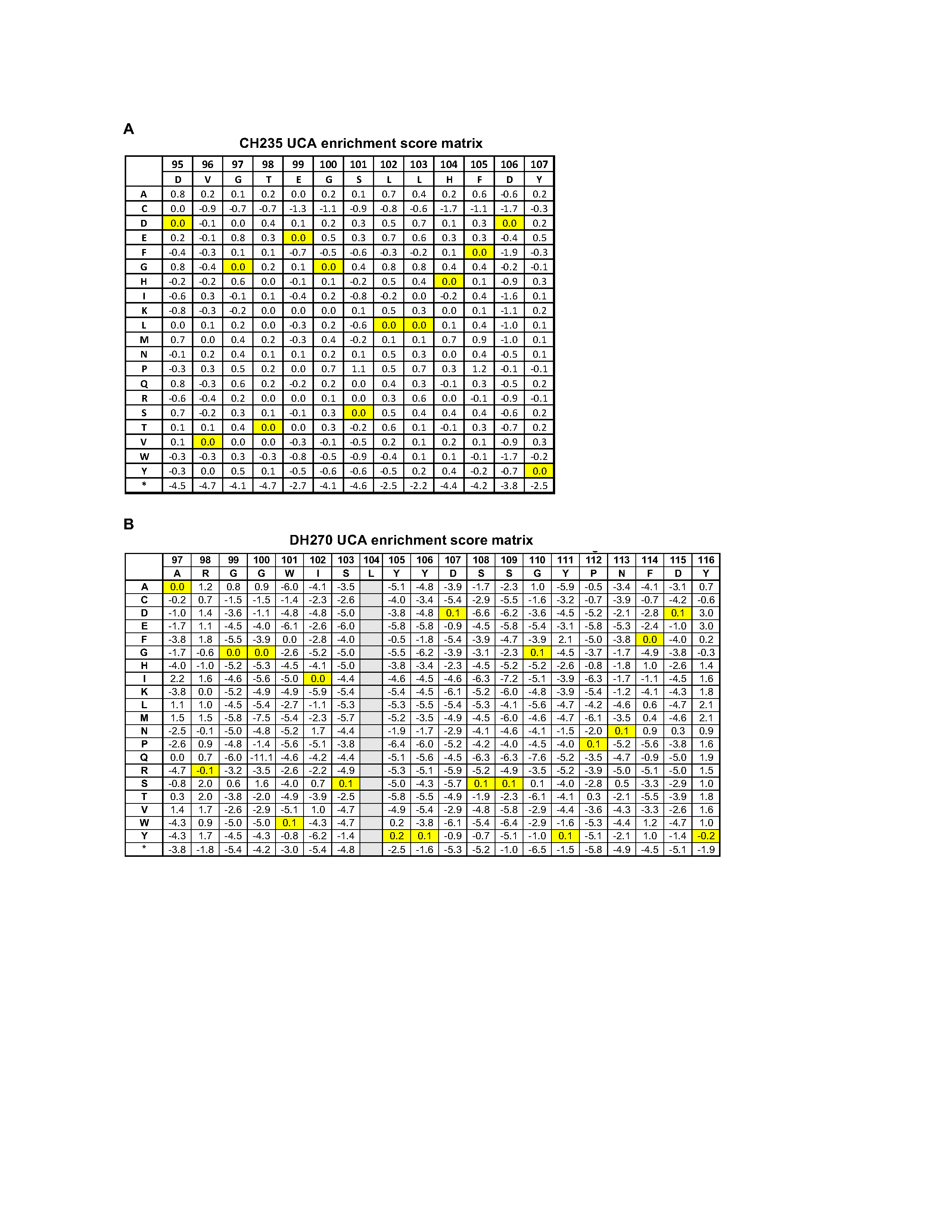

Supplement: S3 Fig — Enrichment scores, calculated as log2 (fold change) of scFv residues before and after sorting with candidate immunogens. A. Enrichment scores for each position of CH235 UCA CDRH3 after selection with immunogen CH505.M5.G458Y. B. Enrichment scores for each position of DH270 UCA CDRH3 after selection with immunogen 10.17DT. Wild type residues shown in yellow. * Stop codon. (TIFF) [file ppat.1011401.s005.tiff]

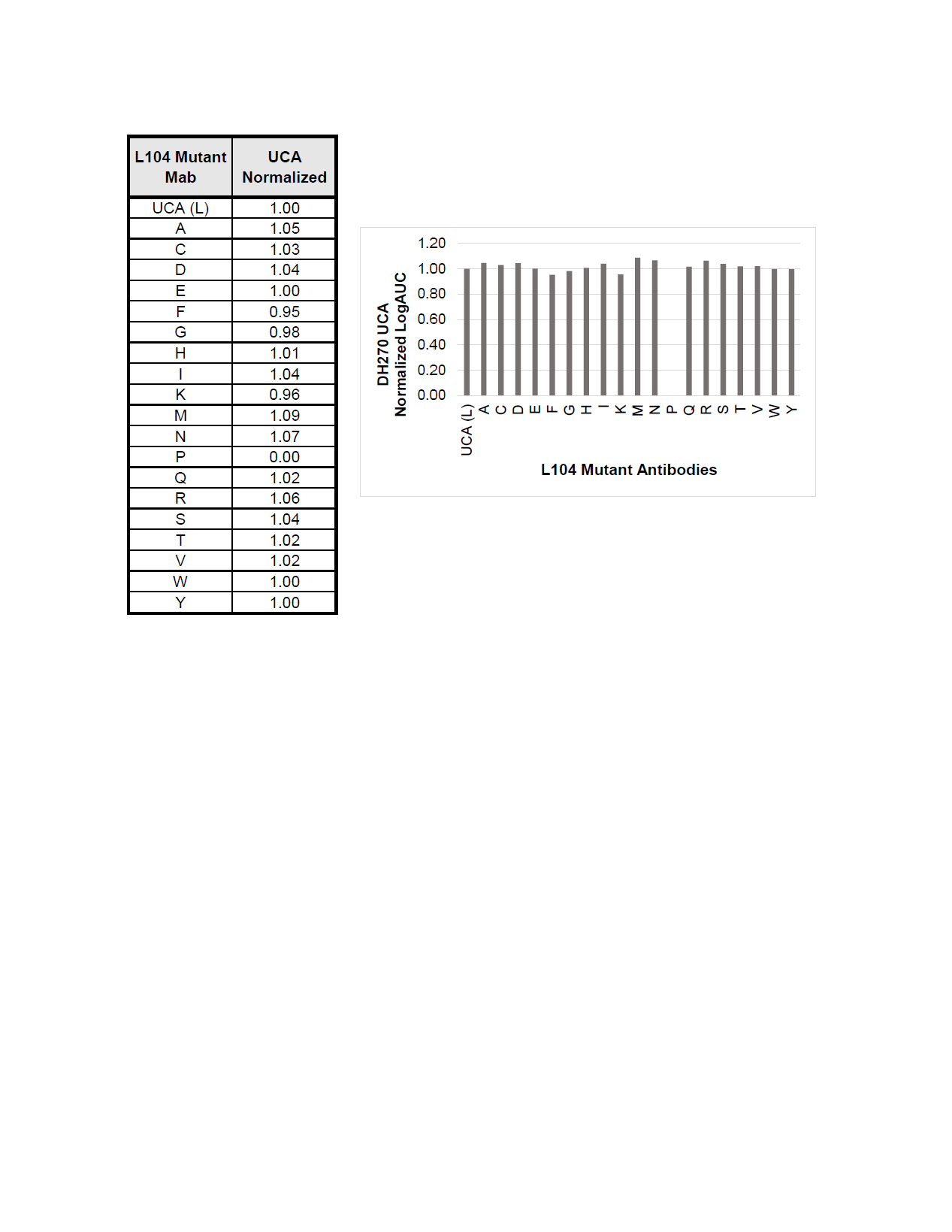

Supplement: S4 Fig — Single site mutated antibodies containing every possible substitution residue at position 104 of DH270 UCA were recombinantly produced and tested for binding to 10.17DT target immunogen. Results are reported as logAUC, normalized to DH270 UCA binding signal. (TIFF) [file ppat.1011401.s006.tiff]

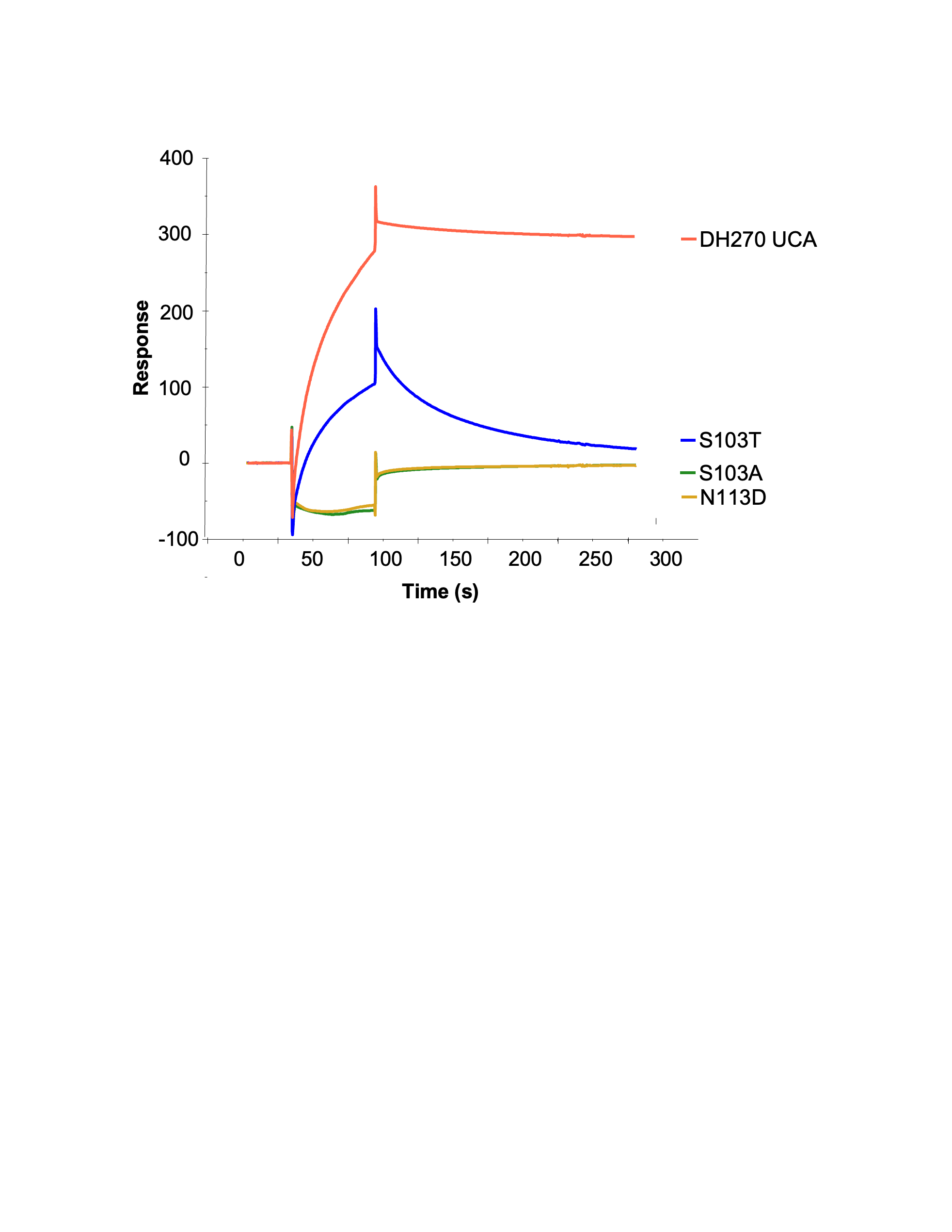

Supplement: S5 Fig — (TIFF) [file ppat.1011401.s007.tiff]

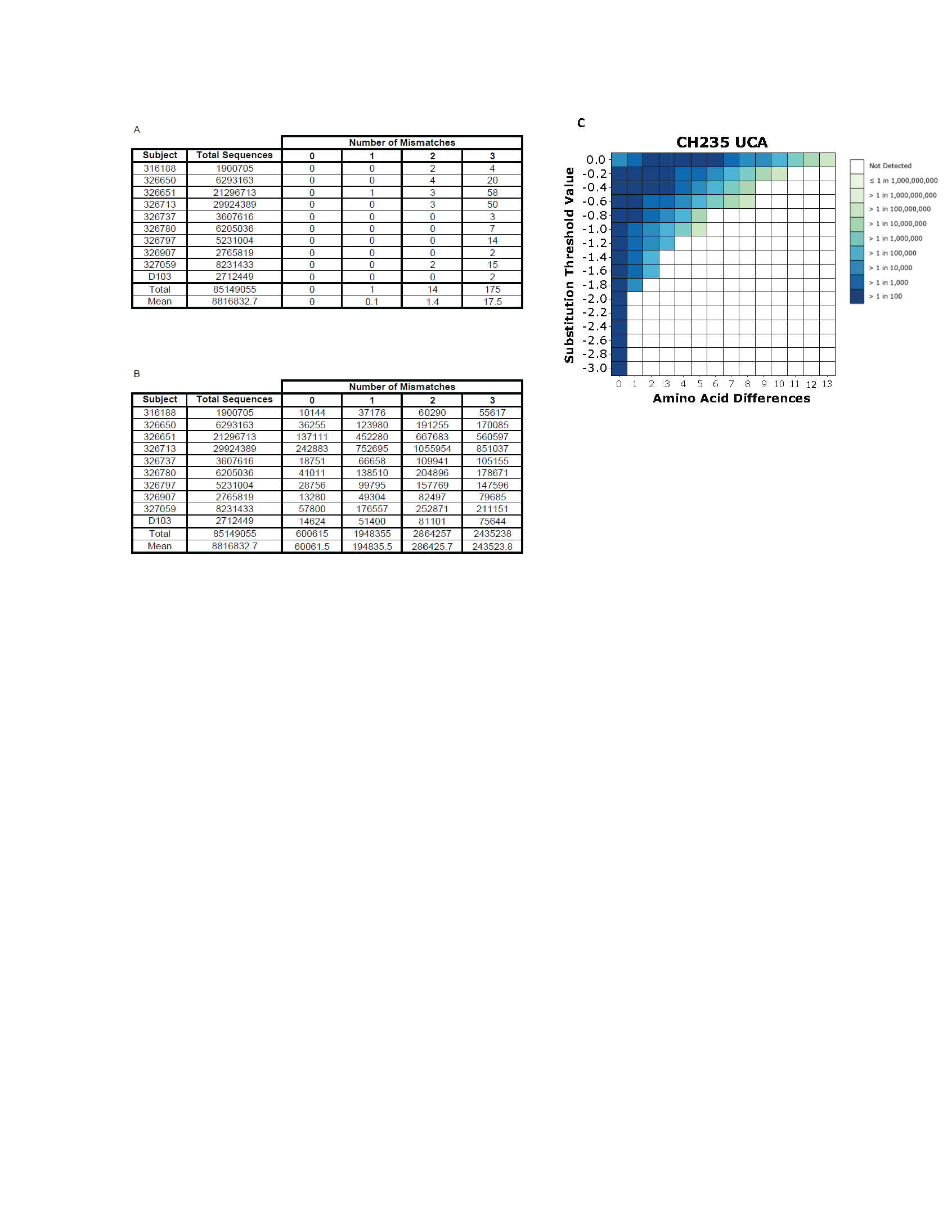

Supplement: S6 Fig — A. The total number of sequences isolated together with the number of CDRH3 sequences that contain 0, 1, 2, or 3 mismatches relative to the DH270 UCA CDRH3 substitution profile recognized by 10.17DT. B. The total number of sequences isolated together with the number of CDRH3 sequences that contain 0, 1, 2, or 3 mismatches relative to the CH235 UCA CDRH3 substitution profile recognized by CH505.M5.G458Y. C. CH505.M5.G458Y compatible (CH235 UCA-like) CDRH3s in experimental BCR sequence database. Heatmap shows the calculated CDRH3 loop frequency for a given number of amino acid differences (x-axis) and allowing for varying degrees of substitution threshold values (y-axis) relative to the sequence of CH235 UCA. Substitution threshold values were determined by deep scanning mutagenesis in Fig 3B. Higher substitution threshold values correspond to a stricter definition of what constitutes a matching amino acid in the CDRH3 substitution profile. (TIFF) [file ppat.1011401.s008.tiff]

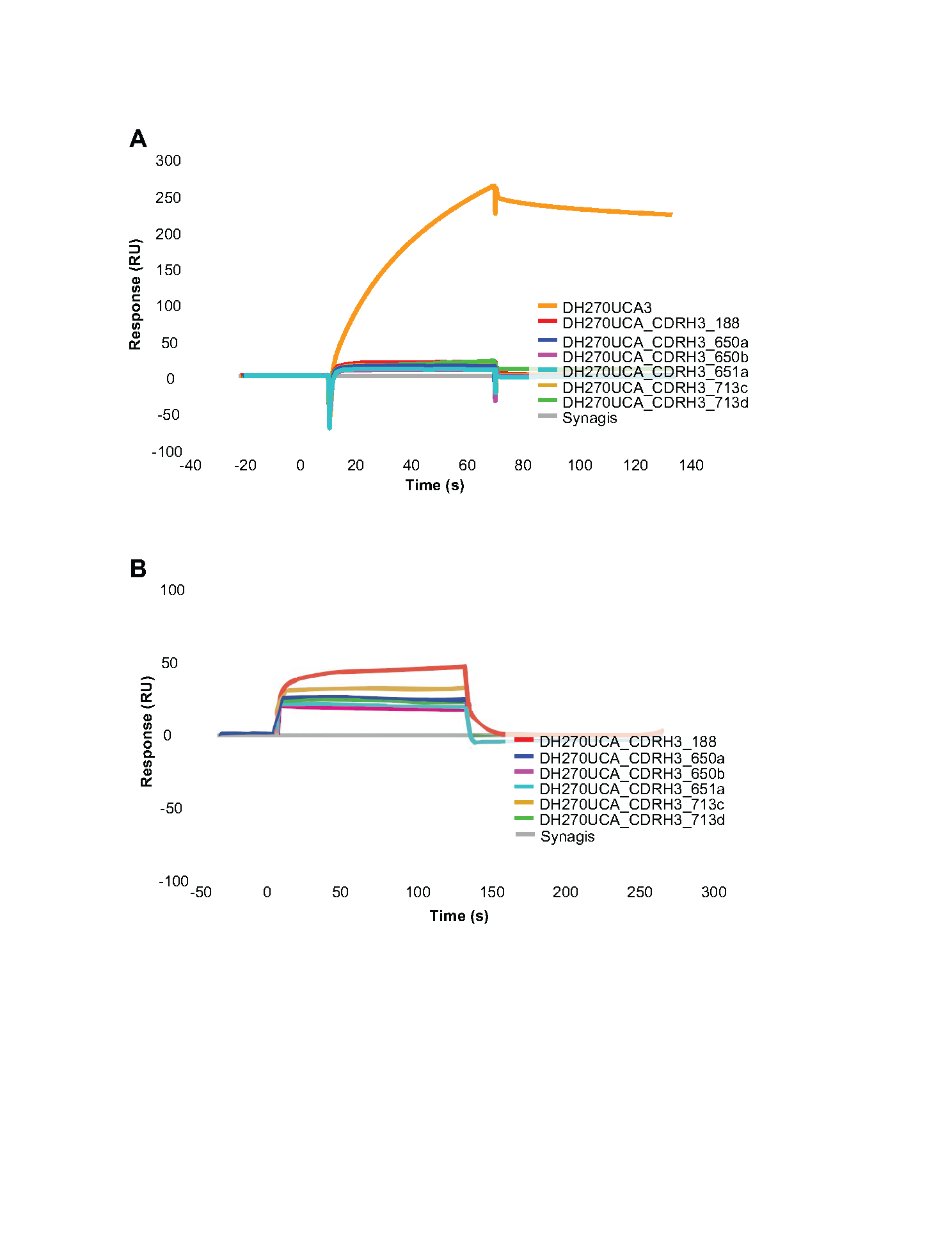

Supplement: S7 Fig — A. 10.17DT SOSIP immobilized on chip (2784 RU) with monoclonal antibodies injected at 12 μM concentration. B. Chip from A with chimeric CDRH3 antibodies and negative control (Synagis) injected at concentration of 20 μM. (TIFF) [file ppat.1011401.s009.tiff]

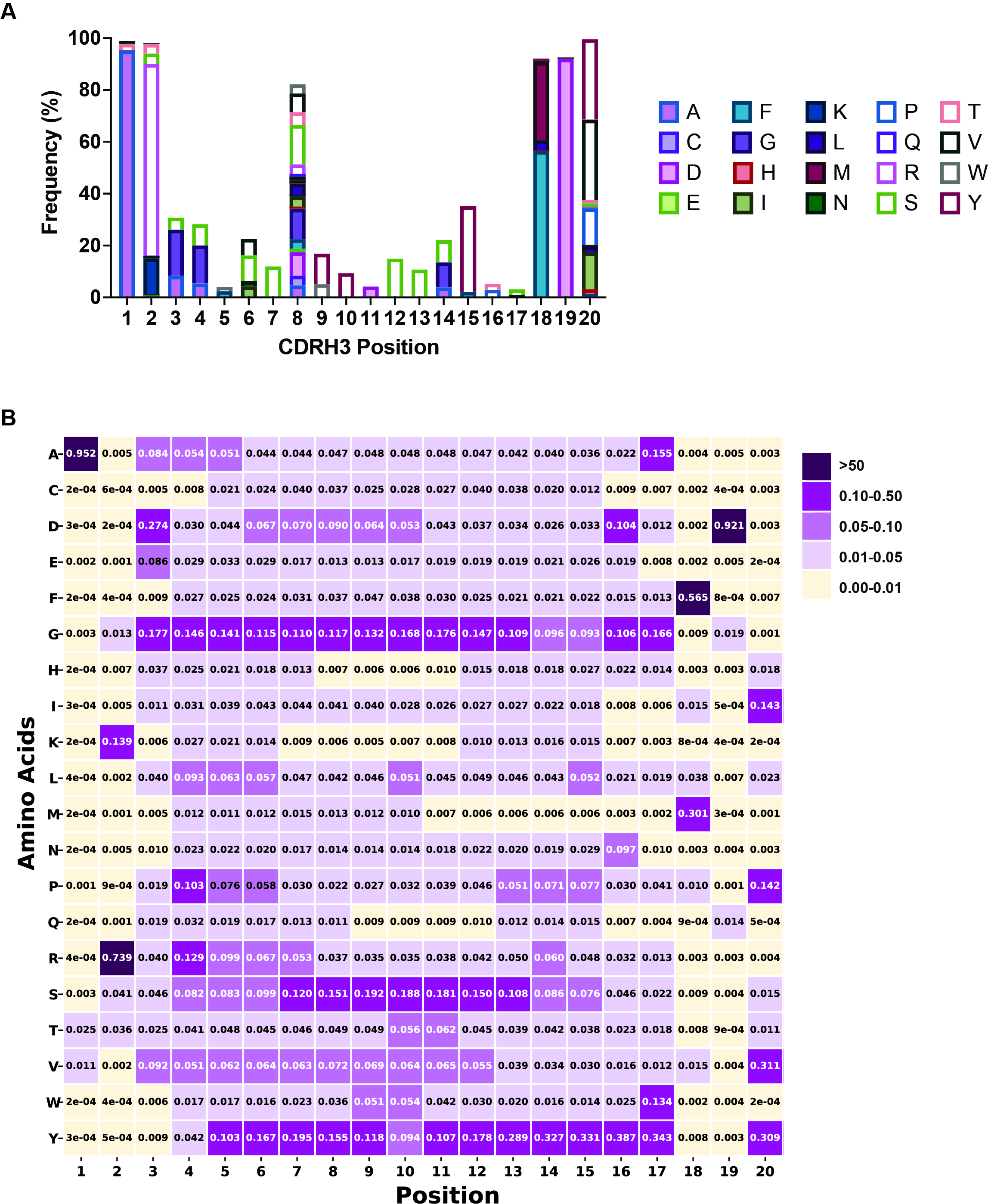

Supplement: S8 Fig — A. The amino acid frequency at each position amongst 20 amino acid long CDRH3 sequences from the BCR database estimated to be compatible with 10.17DT binding (enrichment score > -0.2) in the DH270 UCA CDRH3 substitution profile. Positions 5, 16, and 17 are highly restricted relative to the amino acids observed in these positions in the BCR database. B. The frequency of all amino acids at each position in 20 amino acid long CDRH3s in the BCR database. Immunogens that can bind and tolerate alternative high frequency amino acids at position 5 (e.g. Gly, Tyr, and Ser), at position 16 (e.g. Tyr, Gly, and Asp), and at position 17 (e.g. Tyr, Gly, and Ala) would greatly improve the overall frequency of compatible CDRH3s in the BCR repertoire. (TIF) [file ppat.1011401.s010.tif]
